# Calcium peroxide nanoplatform-mediated cancer immunotherapy enhancement: current advances and perspective

**DOI:** 10.7717/peerj.21115

**Published:** 2026-06-17

**Authors:** Yimin Huang, Lili Xue, Chuanchuan He, Limin Jin

**Affiliations:** Jiaxing Maternity and Child Health Care Hospital, Affiliated Women and Children Hospital, Jiaxing University, Jiaxing, China

**Keywords:** CaO_2_, Nanomaterial, Immunotherapy, TME

## Abstract

The efficacy of cancer immunotherapy is severely constrained by the immunosuppressive tumor microenvironment (ITME), with hypoxia, acidosis, and immune cell dysfunction being key factors contributing to treatment resistance. Calcium peroxide (CaO_2_) nanomaterials leverage their unique hydrolysis behavior within the acidic tumor microenvironment (TME) to simultaneously release oxygen (O_2_), hydrogen peroxide (H_2_O_2_), and calcium ions (Ca^2+^), while consuming hydrogen ions (H^+^). This process synergistically remodels the TME and enhances antitumor immunity through multiple mechanisms: O_2_ alleviates hypoxia, thereby reversing immunosuppression and enhancing the efficacy of O_2_-dependent therapies; H_2_O_2_ catalyzes the production of highly toxic reactive oxygen species (ROS) *via* Fenton or Fenton-like reactions, directly killing tumor cells; H^+^ consumption counteracts acidosis and improves immune cell function; and excessive Ca^2+^ influx induces calcium (Ca) overload, triggering mitochondrial and endoplasmic reticulum (ER) stress to further amplify apoptosis and immune activation. These effects form a multi-pathway, coordinated immunomodulatory network, offering a novel approach to overcome tumor immune resistance. This review summarizes the strategies and mechanisms of CaO_2_ nanoplatforms in cancer immunotherapy, discusses their translational challenges and prospects, and provides insights to advance intelligent nanomedicine for overcoming immunotherapy resistance.

## Introduction

Cancer remains one of the major threats to human life. While conventional treatments, including surgery, radiotherapy, and chemotherapy have achieved considerable success against primary cancers, however, failed in the control of metastasis and recurrence (Mellman, Coukos & Dranoff, 2011; Saxena et al., 2025). Over the past decades, immunotherapy has witnessed revolutionary breakthroughs (Song, Musetti & Huang, 2017), particularly, immune checkpoint inhibitors (ICIs) and chimeric antigen receptor T-cell (CAR-T) therapy have demonstrated outstanding and durable clinical efficacy across a spectrum of malignancies (Chen & Mellman, 2017). Cancer immunotherapy is a therapeutic strategy that utilizes and enhances the function of the immune system to identify, attack, and eliminate tumor cells (Eslami & Lu, 2024). However, its efficacy remains limited in immunologically “cold” tumors, posing a significant challenge in clinical application (Binnewies et al., 2018). Such “cold” tumors are characterized by a highly immunosuppressive tumor microenvironment (ITME), which creates multiple physiological barriers to effective immune activity. For instance, severe hypoxia not only inhibits the function of cytotoxic T lymphocytes (CTLs) and drives their exhaustion but also promotes the accumulation of immunosuppressive cells such as T-regulatory cells (Tregs) and M2-type tumor-associated macrophages (TAMs) (Noman et al., 2015). Concurrently, acidosis caused by abnormal glycolysis in tumor cells leading to excessive lactic acid (LA) production, directly impairs the activity and infiltration ability of immune cells (Vaupel & Multhoff, 2021). Furthermore, dense physical barrier and a lack of effective T-cell chemokines collectively result in insufficient immune cells infiltration (Binnewies et al., 2018). These interconnected mechanisms sustain a state of immune exclusion or suppression, hindering the effectiveness of even excellent immunotherapies (Bagchi, Yuan & Engleman, 2021). This underscores that the key to enhancing the efficacy of immunotherapy lies in overcoming the ITME.

Calcium peroxide (CaO_2_), as a typical oxygen (O_2_)-generating inorganic functional material, has shown prominent promise in cancer therapy (He et al., 2021). Its therapeutic potential stems primarily from its hydrolysis within the tumor microenvironment (TME). Specifically, CaO_2_ reacts with H_2_O and abundant hydrogen ions (H^+^) in the TME to continuously releases O_2_ and hydrogen peroxide (H_2_O_2_) (Wu et al., 2020). The released O_2_ effectively alleviates tumor tissue hypoxia, reverses the immunosuppression, and improves the efficacy of O_2_-dependent cancer therapies, such as chemodynamic therapy (CDT) and photodynamic therapy (PDT) (Liu et al., 2017). H_2_O_2_, as a crucial substrate, catalyzes the generation of highly toxic reactive oxygen species (ROS) *via* Fenton or Fenton-like reactions, which directly induce tumor cell death and provoke immunogenic cell death (ICD) (Tang et al., 2019; Zhang et al., 2020). In addition, the excessive influx of calcium ions (Ca^2+^), a reaction by-product, induces calcium (Ca) overload within tumor cells, which trigger endoplasmic reticulum (ER) and mitochondrial stress, further exacerbating apoptosis and amplifying the ICD effect (Cheng et al., 2006; Bai et al., 2022). Thus, CaO_2_ reshapes the ITME through a synergistic mechanism involving hypoxia alleviation, ROS substrates supply, and Ca overload induction, providing a compelling strategy to potentiate cancer immunotherapy.

**Figure 1:**
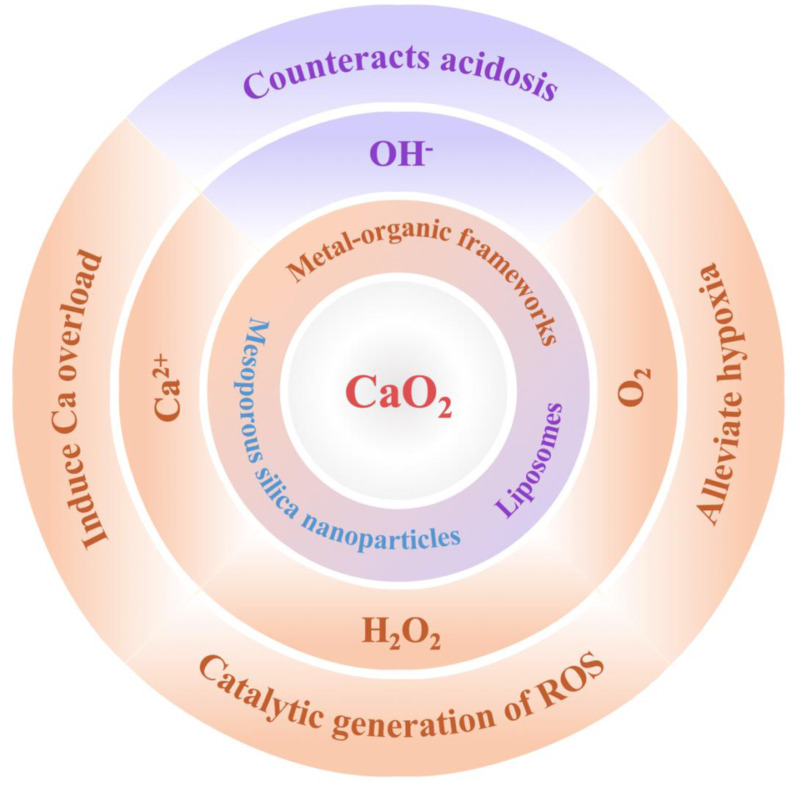
Schematic diagram of the underlying mechanism of CaO_2_ nanoparticle-mediated cancer immunotherapy. Nanoparticles such as liposomes, mesoporous silica nanoparticles, and metal–organic frameworks deliver CaO_2_ to the tumor site. Within the acidic tumor microenvironment, CaO_2_ hydrolyzes to release O_2_, H_2_O_2_, and Ca^2+^, while consuming H^+^. Specifically, the released O_2_ alleviates hypoxia, the generated H_2_O_2_ catalyzes the production of highly toxic ROS, the consumption of H^+^ counteracts acidosis, and the influx of Ca^2+^ induces calcium overload. These effects synergistically remodel the immunosuppressive tumor microenvironment.

Nanomaterials offer new possibilities for drug delivery and immunomodulation owing to their nanoscale size, huge specific surface area, enhanced reactivity, and tunable interfacial properties (Bayda et al., 2019). In recent years, a variety of nanomaterials have been widely employed to construct efficient delivery system for CaO_2_ (Peer et al., 2007). Liposomes, benefiting from their excellent biocompatibility and modifiability, can effectively encapsulate and protect CaO_2_ from premature degradation (Sercombe et al., 2015). Metal–organic frameworks (MOFs), featuring adjustable pore sizes and extremely high specific surface areas, enable high loading capacities and stimuli-responsive release of CaO_2_ in response to TME stimulation (Furukawa et al., 2014). Moreover, mesoporous silica nanoparticles (MSNs), with their ordered pore structure, high loading capacity, and readily functionalized surface, also serve as excellent platforms for the targeted delivery and controlled release of CaO_2_ (Li, Zhang & Feng, 2019). These nanocarriers target and aggregate in tumor tissues through enhanced penetration and retention (EPR) effect or *via* active targeting ligand modifications, subsequently releasing CaO_2_ and its reactive products in response to TME-specific stimuli. This targeted approach maximizes therapeutic efficacy while minimizing systemic toxicity (Peer et al., 2007), thereby establishing a solid foundation for the efficient and safe application of CaO_2_ in cancer immunotherapy. In this article, we comprehensively review recent advances in CaO_2_ nanoparticle-mediated cancer immunotherapy, analyze and elucidate the underlying therapeutic mechanisms (Fig. 1), and provide insights into the current clinical translation challenges along with future prospects for this nanoplatform.

## Survey Methodology

Given that this review aims to synthesize the recent advancements and mechanistic insights into CaO_2_ nanoplatforms for cancer immunotherapy, a systematic and iterative literature search strategy was employed. The primary objective was to capture a comprehensive collection of studies detailing the design, mechanisms, and antitumor immune responses of CaO_2_-based nanotherapeutics.

The literature search was conducted across several major academic databases, including PubMed, Web of Science, Scopus, and the Chinese National Knowledge Infrastructure (CNKI). The search focused on articles published between 2018 and 2024 to ensure the inclusion of the most recent and relevant research, while seminal earlier publications were also considered. The initial search strategy utilized a core set of keywords and their combinations: “nanoparticle”, “CaO_2_ nanoplatform”, “tumor immunotherapy”, “ICD”, “metal immunotherapy”, “ferroptosis”, and “cuproptosis”. As the research progressed and the scope of the literature became clearer, the search strategy was refined and expanded using additional specific keywords to capture the diversity of mechanisms and nanomaterial types. These included: “Ca overload”, “ROS”, “STING agonist”, “TME reprogramming”, “hypoxia alleviation”, “CDT”, “liposomes”, “MOFs”, and “MSNs”.

The initial search yielded over 3,000 publications, and were first filtered to retain studies within the fields of “pharmacology and pharmacy”, “oncology”, “immunology”, and “materials science”. The remaining studies were screened by their titles and abstracts against the following inclusion criteria: (1) Original research articles or reviews explicitly focusing on the synthesis and application of CaO_2_-containing nanoparticles. (2) Studies where the core therapeutic mechanism involved CaO_2_ hydrolysis (*e.g.*, O_2_/H_2_O_2_ production, Ca overload) for cancer treatment. (3) Research that investigated the subsequent immune responses, such as the induction of ICD, activation of dendritic cells (DCs)/T cells, or polarization of TAMs. Following the removal of duplicates and irrelevant articles, 22 key publications were selected as the primary literature for this in-depth review. To ensure comprehensiveness, the reference lists of these selected articles were also traced to identify any additional pertinent documents that may have been missed in the database search.

## CaO_2_ Produce O_2_ to Enhance Immunotherapy

The aberrantly rapid proliferation of tumor tissues leads to a persistent hypoxic microenvironment, which not only promotes malignant progression but also severely suppresses the function of effector immune cells and facilitates the infiltration of immunosuppressive cells (DePeaux & Delgoffe, 2021). As an endogenous O_2_ generator, the accumulation of CaO_2_ at tumor sites directly alleviates hypoxia and reverses immunosuppression (Chen et al., 2019; Ruan et al., 2023). This section will focus on strategies that utilize CaO_2_-mediated O_2_ generation to ameliorate the TME, enhance therapeutic efficacy, and promote immune cell function.

### Potentiating PDT through O_2_ self-supply

The efficacy of numerous effective tumor ablation modalities such as PDT is strictly dependent on local O_2_ concentration (Sun et al., 2025). The hydrolysis of CaO_2_ enables continuous endogenous O_2_ supply for these therapies, thereby elevating the O_2_ partial pressure at the tumor site, significantly strengthening the cytotoxic efficacy of therapies like PDT, and further potentiating the ICD (Garg et al., 2017). For instance, Wei et al. (2025) designed a microneedle patch that utilizes a core CaO_2_ nanoreactor to generate O_2_ intratumorally, which significantly alleviated hypoxia, enhanced the PDT effect of the encapsulated photosensitizer, and intensified ICD through exacerbated ROS storm and mitochondrial damage. In combination with immunomodulatory molecules, this approach induces a down-regulation of M2-type TAMs infiltration, and nearly completely suppressed tumor growth in a melanoma model. However, such microneedle patches represent a local administration strategy, which may have limited applicability for deep-seated or metastasized tumors. Furthermore, the rate of O_2_ generation and its diffusion distance require optimization in more complex models. Similarly, a multilayer core–shell nanoplatform employed an outer CaO_2_ layer to ameliorates hypoxia and enhance the PDT of an internal photosensitizer. In a subcutaneous hepatocellular carcinoma xenograft mouse model, this combined therapeutic strategy achieved a tumor inhibition rate as high as 89.45%, with complete tumor ablation observed within 13 days post-treatment and no recurrence (An et al., 2024a; An et al., 2024b).

For tumors with deep physical or biochemical barriers, such as the mucus layer in colorectal cancer (CRC), passive O_2_ diffusion may be insufficient. Innovatively, Wu et al. (2023) combined the O_2_-generating capability of CaO_2_ with the active motility of nanoparticles. Briefly, under near-infrared (NIR-II) radiation, the O_2_ produced by CaO_2_ acted synergistically with the photothermal effect to drive the CaO_2_-loaded MSNs to penetrate the dense mucus barrier and accumulate effectively in the deep tumor tissue. This platform not only alleviated hypoxia but also significantly increased the proportions of CD4^+^ and CD8^+^ T cells in the spleen (by 1.9-fold and 2.4-fold respectively) and remodeled the gut microbiota. When combined with the immune checkpoint inhibitor αPD-L1, it demonstrated potent efficacy in eradicating primary tumors and inhibiting metastasis in an orthotopic CRC model (Wu et al., 2023). This O_2_-driven penetration mode overcomes the limitation of passive diffusion inherent to traditional nanomedicines, providing a novel insight for the therapy of tumors with deep barriers. Nevertheless, this approach relies on external NIR-II laser irradiation, whose tissue penetration depth is finite and may not be suitable for tumors located too deeply.

### Improving immune cell infiltration and function

Hypoxia within the TME directly impairs the cytotoxic function of CD8^+^ T cells, promotes their exhaustion by pathways such as hypoxia-inducible factor-1α (HIF-1α) activation, and recruits immunosuppressive cells like Tregs (Watson et al., 2021). Alleviating tumor hypoxia *via* CaO_2_ can directly create a more favorable microenvironment for immune cells (Smith et al., 2023). For example, as self-triggered thermoelectric nanoheterojunction (BST/CaO_2_) hydrolyzes in the TME to generate O_2_ and heat, thereby driving the generation of ROS. Concurrently, the released Ca^2+^ was shown to promote DC maturation and tumor antigen presentation. In a CRC xenograft mouse model, this treatment increased the proportion of mature DCs by 4.1-fold compared to the control and significantly elevated serum levels of inflammatory cytokines interferon-γ (IFN-γ) and tumor necrosis factor-α (TNF-α), indicating systemic activation of anti-tumor immunity (Yuan et al., 2023). This study highlights the potential of CaO_2_-induced O_2_ generation to indirectly enhance antigen presentation and activate adaptive antitumor immunity. It is important to note that most current evidence originates from murine models, whether human immune cells respond identically to CaO_2_-mediated microenvironmental alterations awaits validation in clinical studies.

## CaO_2_ Produce H_2_O_2_ to Enhance Immunotherapy

H_2_O_2_ serves as an essential reactive substrate for catalytic therapeutic strategies, especially CDT. However, the efficacy of these therapies is often constrained by the insufficient endogenous levels of H_2_O_2_ within tumors, limiting the initiation of a robust therapeutic response (Tang et al., 2019). Ingeniously, this critical challenge of substrate deficiency is effectively addressed by CaO_2_, which undergoes hydrolysis in the acidic TME to provide a sustained, localized release of H_2_O_2_ (Shi et al., 2023). In this section, the therapeutic strategies that utilize CaO_2_-mediated H_2_O_2_ generation as an energy source for catalytic reactions is focused. It details the role of CaO_2_ as an *in-situ* H_2_O_2_ reservoir and how it drives a cascade of catalytic processes. The resultant generation of highly ROS induces intense oxidative stress, which not only directly damages tumor cells but also serves as a key danger signal to provoke and potentiate antitumor immune response (Zhang et al., 2019).

### Amplifying CDT and ferroptosis

CDT operates on the principle of converting H_2_O_2_ into highly toxic hydroxyl radicals (⋅OH) *via* Fenton or Fenton-like reactions. The self-supplying H_2_O_2_ characteristic of CaO_2_ significantly augments this process, making it one of the most widely adopted strategies to boost CDT efficacy (Meng et al., 2020; Liu et al., 2021). A typical design is the CCZC nanoplatform composed of curcumin (Cur)-coated CaO_2_@ZIF-Cu nanoparticles. Upon disintegration within the TME, CCZC releases copper ions (Cu^2+^) and exposes CaO_2_. The H_2_O_2_ generated from CaO_2_ hydrolysis immediately reacts with Cu^2+^ in a Fenton-like reaction, markedly producing ⋅OH for potent CDT. In a melanoma model, this achieved a 78% tumor inhibition rate while significantly promoting DC maturation and CTLs proliferation (Mo et al., 2024). However, the Fenton reaction efficiency of Cu^2+^ in the weakly acidic TME is relatively lower compared to ferrous irons (Fe^2+^), and the long-term biosafety of Cu^2+^ requires further evaluation. Another design (Cu_2_Se–CaO_2_@LA) utilized NIR-II laser to control CaO_2_ exposure, generating H_2_O_2_ in-situ for photothermally-enhanced CDT. This acted synergistically with Ca overload to induce mitochondria and ER stress *via* the ROS–PERK–eIF2α signaling pathway, triggering potent ICD, promoting DC maturation, increasing the infiltration of CD4^+^ and CD8^+^ T cells within tumors, elevating serum levels of inflammatory cytokines such as TNF-α, interleukin-6 (IL-6), and IFN-γ, and achieving an 83% inhibition rate in a breast cancer model (Feng et al., 2023). Liang et al. (2023) further integrated the functions of the hydrolysis products of CaO_2_ within a MOF platform. They designed a hyaluronic acid (HA)-targeted, copper-doped ZIF-8 nanoparticle (denoted as HA/CaO_2_-Ce6@Cu-ZIF) for the co-delivery of CaO_2_ and the photosensitizer Ce6, which enhanced CDT *via* a Fenton-like reaction between the self-supplied H_2_O_2_ and Cu^2+^, significantly improved the PDT efficacy of Ce6 by using the simultaneously generated O_2_ to alleviate hypoxia, and synergistically aggravated tumor cell death through induced Ca overload. In the Panc02 tumor-bearing mice model, this strategy achieved a tumor inhibition rate of 60.80% without inducing significant systemic toxicity, demonstrating its potential as a self-supplying nanoreactor for synergistic catalytic therapy.

Furthermore, H_2_O_2_ can be utilized to induce ferroptosis and synergize with immunomodulation. For example, an iron-based MOF nanoplatform employed CaO_2_-generated H_2_O_2_ to engage in a Fenton reaction with reduced Fe^2+^, initiating ferroptosis. Concurrently, co-loaded oridonin (ORI) inhibited two key negative regulatory pathways, HSPB1/PCBP1 and FSP1/CoQ10, synergistically enhancing ferroptosis. *In vivo* experiments revealed its tumor suppression effect in melanoma that was 5.6 times higher than CDT alone, and it worked synergistically with fusion membrane (FM)-mediated immunotherapy (Cai et al., 2024). Similarly, a pH-responsive membrane-coated nanozyme system co-loaded with CaO_2_ and ferriferrous oxide (Fe_3_O_4_) generated H_2_O_2_ from CaO_2_ in the TME, which was subsequently catalyzed by Fe_3_O_4_ nanoparticles in a Fenton-like reaction to produce ⋅OH and induce ferroptosis. The accompanying pH change was also shown to induce the repolarization of TAMs, achieving a synergistic effect between ferroptosis and immunomodulation in a breast cancer model, significantly prolonging mouse survival with 100% survival at day 80 (Li & Rong, 2020). Feng et al. (2025) further complexed CaO_2_ with ferric ions (Fe^3+^)-doped dendritic mesoporous organosilica (DMOS). In this system, the Fenton reaction initiated ferroptosis and ICD, elevated the maturation rate of splenic DCs to 38.10%, and enhanced local and systemic infiltration of both CD8^+^ and CD4^+^ T cells. When combined with anti-PD-1 therapy, the inhibition rate of primary breast cancer reached 89.10%, and the proportion of memory T cells in the spleen reached 43.60%, indicating that this therapy has stimulated long-term immune memory.

More sophisticated strategies aim to simultaneously induce multiple cell death modalities. For instance, a cascade nanoreactor utilized CaO_2_-produced H_2_O_2_ to enhance Cu^2+^-based CDT, while Ca overload exacerbated oxidative stress. Excessive Cu^2+^ could both initiate cuproptosis and deplete GSH and accumulate lipid peroxide (LPO) to synergistically induce ferroptosis. This CaO_2_-driven activation of multiple cell death modalities robustly stimulates ICD, effectively converting “cold” tumor into “hot” tumor in a breast cancer mouse model, achieving a 68.10% tumor inhibition rate, significantly promoting the maturation of DC, and increasing the proportion of intratumoral CD8^+^ T by 2.3-fold compared to the control (Tang et al., 2025). However, this cascade nanoreactor is overly complex, its dominant mechanism and the intersections among various pathways are difficult to precisely control *in vivo*, posing challenges for clinical translation. Moreover, the concurrent induction of cuproptosis and ferroptosis may lead to enhanced toxicity, necessitating more stringent safety evaluations regarding potential effects on normal tissues.

### Synergizing with other catalytic modalities

Based on the foundation of Fenton reaction, the utility of H_2_O_2_ extends beyond CDT to potentiate other modalities such as sonodynamic therapy (SDT) (Du et al., 2023). Building on the concept of multi-modal enhancement, the CTFAP nanocomposite utilized H_2_O_2_ supplied by CaO_2_, which acted both as a substrate for the sonosensitizer tetracarboxylic porphyrin under ultrasound to enhance SDT, and participated in the Fe^3+^-mediated Fenton-like reaction to synergistically initiate ferroptosis. Remarkably, this design established a self-reinforcing ferroptosis positive feedback loop driven by IFN-γ secreted by activated CTLs upregulation of ACSL4, which subsequently accelerated lipid peroxidation (Jiang et al., 2024). Despite the remarkable efficacy, controlling the intensity of such self-reinforcing loops remains challenging, and the potential risk of systemic inflammation requires attention in follow-up studies.

**Figure 2:**
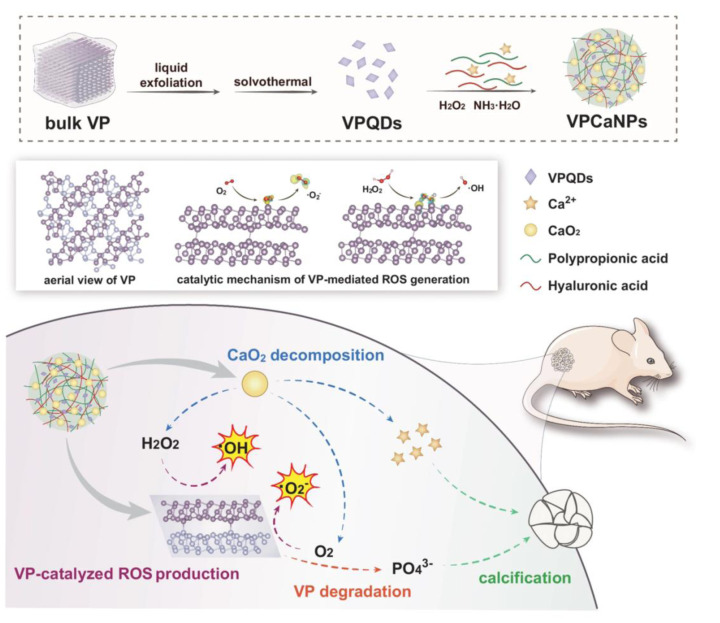
Schematic illustration of the preparation and antitumor mechanism of VPCaNPs. The preparation of VPCaNPs contains the top-down synthesis of VPQDs and the one-pot fabrication of VPCaNPs. The antitumor properties are based on the fate of VPCaNPs, including the release of cargos, CaO_2_ decomposition, VP-catalyzed ROS generation, O_2_-mediated VP degradation, and calcification. Image source credit: https://www.nature.com/articles/s41467-024-50769-0/figures/1, CC BY-NC-ND 4.0.

In exploring novel catalytic mechanisms, Zhang et al. (2024) introduced violet phosphorus quantum dots (VPQDs) as an exogenous-stimulus-free catalyst. They utilized the O_2_ and H_2_O_2_ continuously supplied by CaO_2_ decomposition as reaction substrates, overcoming the scarcity of substrate in tumors and effectively triggering oxidative stress-mediated apoptosis. Interestingly, the phosphate by-products generated in this process synergized with Ca^2+^ to promote tumor calcification in a 4T1 breast cancer model (Fig. 2), revealing a novel functional role for therapeutic metabolites. However, as a novel nanomaterial, the long-term biocompatibility of VPQDs and the clearance mechanisms of their degradation products *in vivo* require systematic investigation.

## CaO_2_ consume H^+^ to enhance immunotherapy

The metabolic preference of tumor cells for glycolysis leads to substantial LA accumulation, resulting in an acidic TME that impairs immune function through multiple mechanisms (Watson et al., 2021). A low extracellular pH (typically pH 6.5−6.9) has been shown to inhibit T-cell receptor signaling and impair glycolytic metabolism essential for T cells (Calcinotto et al., 2012). Similarly, the cytolytic activity and IFN-γ production of natural killer (NK) cells are significantly dampened under acidic conditions (Harmon et al., 2019). By consuming H^+^, CaO_2_ hydrolysis increases the local pH, thereby alleviating this direct inhibition and potentially restoring the metabolic fitness and cytotoxic potential of tumor-infiltrating lymphocytes (Wang et al., 2022).

The ability of CaO_2_ to consume H^+^ and neutralize acidity can directly ameliorate the ITME. Qiu et al. (2024) developed a zinc-coordinated lipid nanoparticle co-delivering CaO_2_ and the STING agonist diABZI-2. In the acidic TME, CaO_2_ consumed H^+^ and release O_2_, directly ameliorating acidosis and creating favorable conditions for the effective activation of the STING pathway. Simultaneously, zinc ion (Zn^2+^) and Ca^2+^ work in concert to induce robust ICD. The combination of a rectified TME, STING pathway activation, and ICD ultimately leads to a potent, systemic antitumor immune response. In CT26 CRC model, this treatment resulted in an increase in splenic CD8^+^ T cells to 11.30%, promoted DC maturation by more than 1.5-fold, and elicited long-term immune memory. However, the neutralization effect may be inconsistent due to the uneven acidity distribution of TME.

A more profound strategy aims to eliminate the root cause of acidity, Fang et al. (2025) constructed a sophisticated multi-channel immune nano-regulator. Specifically, ultrasound triggered O_2_ generation from perfluorocarbon chains, accelerating H^+^ consumption. Crucially, this strategy not only directly elevated pH and relieved hypoxia but also inhibited glycolysis and LA production, thereby reversing LA-driven M2-type TAM polarization and the functional suppression of CTLs and NK cells. This led to comprehensive remodeling of the ITME, resulting in powerful antitumor immunity and significantly prolonged survival in both breast cancer cellular and animal models. While this strategy intervenes at the metabolic root, its complex nanostructure presents challenges for large-scale production and quality control.

## CaO_2_ produce Ca^2+^ to enhance immunotherapy

The high concentration of Ca^2+^ generated from the hydrolysis of CaO_2_ induces lethal Ca overload within tumor cells. This serves not only as an effective direct cytotoxic mechanism but also as a potent inducer of ER and mitochondrial stress, efficiently triggering ICD (Liu et al., 2025a; Liu et al., 2025b). As a pivotal second messenger, the disruptive influx of Ca^2+^ disrupts cellular homeostasis, directly leading to ICD and concurrently modulating the function of various immune cells (Cheng et al., 2025; Wang et al., 2025). For instance, Ca signaling is integral to T cell receptor (TCR) activation and nuclear factor of activated T cells (NFAT) signaling, which governs T cell proliferation and effector functions (Feske, Wulff & Skolnik, 2015). Thus, the localized flux of Ca^2+^ from CaO_2_ hydrolysis may potentially lower the activation threshold for adjacent T cells. The dual role of Ca^2+^, simultaneously killing tumor cells in an immunogenic manner and potentially enhancing the activity of adjacent immune effectors, highlights its unique value in designing combination immunotherapy strategies.

### Inducing ICD *via* Ca dyshomeostasis

Ca overload rapidly induces severe ER stress, leading to the surface exposure of calreticulin (CRT) and the release of damage-associated molecular patterns (DAMPs) such as ATP and high-mobility group box 1 (HMGB1), making it an effective means to induce ICD (Krysko et al., 2012). Ingeniously, Li et al. (2025) combined the CaO_2_ with irreversible electroporation (IRE). The introduction of Ca^2+^ from CaO_2_ exacerbated the ER stress initiated by IRE, thereby intensifying ICD signaling. Furthermore, in CRC tumor-bearing mice, the vermiculite nanosheet carrier used in this platform adsorbed tumor antigens and DAMPs, forming an in-situ vaccine that dramatically promoted DC activation and antigen cross-presentation. Huang et al. (2022) employed CaO_2_ to design a bidirectional enhancement ICD nanoplatform for breast cancer, in which CaO_2_ hydrolysis-induced Ca overload synergized with copper sulphide (CuS)-based PDT, leading to exacerbated mitochondrial damage and stronger ICD response. Concurrently, the mitochondrial DNA released due to PDT-induced damage was shown to reprogram TAMs towards an anti-tumor M1 phenotype.

Ca overload can also be linked with other therapies to create complex metabolic interference and cell death. For example, a self-reinforcing bimetallic mitochondrial disrupter that intricately links Ca overload with chemotherapy and cuproptosis. In this system, Ca overload synergized with the chemotherapeutic agent doxorubicin (DOX) to exacerbate the ROS storm and inflict severe mitochondrial damage, leading to ATP depletion. The ensuing energy crisis directly impaired copper (Cu) transporter function, causing Cu^2+^ accumulate within mitochondria and thereby sensitizing the breast cancer cells to cuproptosis (Du et al., 2024).

### Modulating immune cell activity through Ca signaling

Ca^2+^ serves not only as a death signal for tumor cells but also as a critical second messenger for immune cell activation. Fluctuations in extracellular Ca^2+^ concentration or the use of Ca^2+^ carriers can directly influence the maturation and antigen-presentation capacity of DCs (Saez et al., 2018). The NVscp nanovaccine leveraged CaO_2_ to rapidly generate ROS and release Ca^2+^ within the endo/lysosomes of antigen-presenting cells (APCs), directly stimulating APC maturation and enhancing antigen cross-presentation. This induced a robust antigen-specific CD8^+^ T cell response and increased the ratio of CD8^+^ T/Treg in a melanoma model (Su et al., 2021). The immunomodulatory effect of Ca^2+^ can also be achieved indirectly by influencing tumor cell metabolism. For instance, EL4 cell membrane-coated nano-CaO_2_, upon targeting tumors, induced Ca overload that inhibited glycolysis in breast cancer cells, thereby reducing the secretion of LA, which in turn contributed to reversing CTLs exhaustion (An et al., 2024a; An et al., 2024b), offering a unique metabolic perspective for Ca-based immunotherapy.

In addition, the immunomodulatory potential of Ca^2+^ extends to synergistic interactions with other metal ions. For example, the HMnO_2_@CaO_2_ nanoplatform utilized Ca overload to cause ER stress and mitochondrial damage in an osteosarcoma mouse model, while the released manganese ions (Mn^2+^) induced ferroptosis and promoted the repolarization of TAMs towards the anti-tumor M1 phenotype (Zhang et al., 2025). The (CaO_2_/TA)-(Fe^3+^/BSA) self-enhancing nanoparticle synergized Ca overload with an intensified Fenton reaction based on Fe^3+^ self-reduction and H_2_O_2_ self-supply, synergistically inducing ferroptosis and ICD. This profoundly reshaped the immune microenvironment in a breast cancer model, as evidenced by increased proportions of mature DCs, M1-type macrophages, and intratumoral CD8^+^ T cells to 51.50%, 41.10%, and 45.60%, respectively, while significantly suppressing immunosuppressive cells like Tregs and myeloid-derived suppressor cells (MDSCs) (Zheng et al., 2024).

Intriguingly, Ca^2+^ signaling can be harnessed for temporal regulation of immune responses. The FePpy@CaO_2_-modified bone implant material utilized Ca^2+^ to participate in activating pro-inflammatory M1 phenotype macrophages during the bone tumor treatment phase. Following tumor eradication, it adaptively promoted macrophage switch to the pro-repair M2 phenotype and enhanced osteogenesis, achieving spatiotemporally precise control of the immune response to integrate therapy with tissue regeneration (Liu et al., 2025a; Liu et al., 2025b).

## Conclusion and Outlook

CaO_2_ nanoplatforms have emerged as a highly promising sensitization strategy for cancer immunotherapy, with their unique TME responsive hydrolysis characteristics enable simultaneously generation of O_2_, H_2_O_2_, and Ca^2+^ (Tang et al., 2019). The advantages and innovations of CaO_2_ nanoplatform in cancer immunotherapy point to their outstanding multi-functional integration capabilities, including alleviating hypoxia, neutralizing acidosis, inducing Ca overload through a single nanoplatform. In addition, achieving synergistic amplification of immunotherapy effects by coordinating sonodynamic, photodynamic, or chemokinetic therapy and delivering immune agonists or chemotherapy drugs (Table 1) (Liu et al., 2019). More importantly, the CaO_2_-based nanotherapeutic strategies have successfully promoted the exploration of metal immunotherapy. The H_2_O_2_ generated from CaO_2_ hydrolysis involved into Fenton or Fenton-like reactions mediated by metal ions such as Cu^2+^ and Fe^3+^, leading to the production of ⋅ OH. This process amplifies intracellular ROS levels, induces mitochondrial damage, and directly modulates the functions of immune cells (Chen et al., 2022; Fan et al., 2025; Liu et al., 2025a; Liu et al., 2025b). Furthermore, CaO_2_ effectively initiated novel regulatory cell death (RCD) like ferroptosis and cuproptosis (Jiang, Stockwell & Conrad, 2021; Tsvetkov et al., 2022), providing a new approach to overcome apoptotic resistance.

**Table 1:** The effects of CaO_2_ combine with different therapeutic strategies on tumor cells.

Therapeutic strategy	Biological impact	Immunological impact	Representative literature
CaO_2_	1. Hydrolysis consumes H^+^, rapidly increasing extracellular pH; 2. Generates H_2_O_2_, leading to elevated intracellular ROS levels; 3. Ca overload causes endoplasmic reticulum and mitochondrial dysfunction; 4. Produces O_2_ to alleviate tissue hypoxia.	1. Reverses immunosuppression of immune cells caused by acidosis; 2. Induces baseline level of ICD.	/
Combined with SDT	CaO_2_ hydrolysis provides H_2_O_2_ as a substrate for sonosensitizers. Under ultrasound irradiation, it synergistically generates massive ROS, which cooperates with Ca overload to cause severe mitochondrial and ER damage.	Potent oxidative stress and organelle damage synergistically induce robust ICD, releasing tumor antigens and activating innate immunity.	Jiang et al. (2024) and Tang et al. (2025)
Combined with CDT	CaO_2_ acts as a self-supplying H_2_O_2_ reservoir, continuously providing substrate for Fe^3+^/Cu^2+^-mediated Fenton/Fenton-like reactions, leading to burst ROS accumulation and directly driving ferroptosis.	DAMPs released from ferroptosis effectively activate dendritic cells, remodel the immunosuppressive microenvironment, and synergize with immune checkpoint blockade.	Feng et al. (2023), Li & Rong (2020), Mo et al. (2024) and Feng et al. (2025)
Combined with PTT/PDT	1. O_2_ generated by CaO_2_ directly alleviates tumor hypoxia, overcoming the oxygen-dependent limitation of PDT; 2. Heat from PTT can trigger controlled release of CaO_2_ and synergize with Ca overload for killing.	Improved oxygenation combined with multiple stressors promotes potent ICD and enhances immune cell infiltration.	Wu et al. (2023), Huang et al. (2022), Wei et al. (2025) and Cai et al. (2024)
Combined with immune adjuvants	Remodels the TME such as neutralizing acidity, inhibiting lactate, and induces ICD, creating a foundation for immune modulation.	Immune adjuvants like TLR agonist R848 are used to reprogram macrophages, thereby directly modulating immune cells.	Fang et al. (2025)

**Notes.**

Ca calcium CaO_2_ calcium peroxide CDT Chemodynamic therapy Cu^2+^ copper ions DAMPs damage-associated molecular patterns Fe^3+^ ferric ions H^+^ hydrogen ions H_2_O_2_ hydrogen peroxide ICD immunogenic cell death O_2_ oxygen PDT Photodynamic therapy PTT Photothermal therapy ROS reactive oxygen species SDT Sonodynamic therapy TME tumor microenvironment

However, the clinical translation of CaO_2_ nanoplatforms remain faces severe challenges. (1) The chemical instability of CaO_2_ imposes stringent requirements for its production and long-term storage (Mitragotri et al., 2015). (2) The long-term biological safety and metabolic clearance pathways of the excess reactive products like ROS generated by the hydrolysis of CaO_2_ *in vivo* remain unclear (Nel et al., 2006). (3) Current studies remain at the preclinical stage, lacking large-scale animal experiments and clinical data to verify the efficacy and toxicity. (4) The individual differences in immune responses caused by tumor heterogeneity pose substantial difficulties in predicting therapeutic outcomes and designing personalized applications.

Therefore, in the future, it is necessary to develop more stable CaO_2_ encapsulation technologies and intelligent stimulus–response release systems to enhance the delivery efficiency and safety. Secondly, elucidate the mechanisms by which CaO_2_ nanoplatforms regulate the TME and activate immunity, potentially by integrating multi-omics techniques. Finally, explore combination strategies with pioneering immunotherapies such as CAR-T, mRNA vaccines, and bispecific antibodies to exert synergistic effects. And ultimately, advancing systematic safety evaluations of CaO_2_ nanoplatform in large-scale animal models to facilitate early-phase clinical trials, thereby validating their clinical potential and achieving translational breakthroughs.

## Abbreviations

APCs: antigen-presenting cells
Ca: calcium
Ca^2+^: calcium ions
CaO_2_: calcium peroxide
CAR-T: chimeric antigen receptor T-cell
CDT: chemodynamic therapy
CNKI: Chinese National Knowledge Infrastructure
CRC: colorectal cancer
CRT: calreticulin
CTLs: cytotoxic T lymphocytes
Cu: copper
Cu^2+^: copper ions
Cur: curcumin
CuS: copper sulphide
DAMPs: damage-associated molecular patterns
DCs: dendritic cells
DMOS: dendritic mesoporous organosilica
DOX: doxorubicin
EPR: enhanced penetration and retention
ER: endoplasmic reticulum
Fe^2+^: ferrous irons
Fe^3+^: ferric ions
FM: fusion membrane
Fe_3_O_4_: ferriferrous oxide
GSH: glutathione
H^+^: hydrogen ions
HA: hyaluronic acid
HIF-1α: hypoxia-inducible factor-1α
HMGB1: high-mobility group box 1
H_2_O_2_: hydrogen peroxide
ICD: immunogenic cell death
ICIs: immune checkpoint inhibitors
IFN-γ: interferon-γ
IL-6: interleukin-6
IRE: irreversible electroporation
ITME: immunosuppressive tumor microenvironment
LA: lactic acid
LPO: lipid peroxide
MDSCs: myeloid-derived suppressor cells
Mn^2+^: manganese ions
MOFs: metal–organic frameworks
MSNs: mesoporous silica nanoparticles
NFAT: nuclear factor of activated T cells
NIR-II: near-infrared
NK: natural killer
O_2_: oxygen
^1^O_2_: singlet oxygen
⋅OH: hydroxyl radicals
ORI: oridonin
PDT: photodynamic therapy
RCD: regulatory cell death
ROS: reactive oxygen species
SDT: sonodynamic therapy
TAMs: tumor-associated macrophages
TCR: T cell receptor
TME: tumor microenvironment
TNF-α: tumor necrosis factor-α
Tregs: T-regulatory cells
VMT: vermiculite nanosheets
VPQDs: violet phosphorus quantum dots
Zn^2+^: zinc ion

## Additional Information and Declarations

### Data Availability

The following information was supplied regarding data availability: This is a literature review.
